# Family Dementia Caregivers’ Disaster Preparedness in Chinese American Communities in New York City

**DOI:** 10.1093/geroni/igaf122.420

**Published:** 2025-12-31

**Authors:** Weiyu Mao, Bei Wu, Yaolin Pei, Zhen Cong

**Affiliations:** University of Nevada, Reno, Reno, Nevada, United States; NYU Shanghai, Shanghai, China (People’s Republic); The University of Texas at Austin, Austin, Texas, United States; Chapman University, Orange, California, United States

## Abstract

Disaster preparedness is a significant public health concern. Disasters manifest in different forms and often lead to disruption of daily life. This is especially challenging for family dementia caregivers and their care recipients in the community. There remains much unknown regarding dementia caregiver disaster preparedness, especially in ethnic and racial communities. Guided by Protection Motivation Theory, with the backdrop of Hurricane Ida, this study explored the association between caregiving-related characteristics, cognitive appraisals, and disaster preparedness among caregivers in Chinese American communities. Data came from a pilot study on Chinese American family dementia caregivers in New York City between November 2021 and June 2022. Purposive sampling was used to recruit caregivers to participate in a survey (online or telephone). Current caregivers (n = 76) were included. The dependent variable was caregiver disaster preparedness, measured by preparedness for Hurricane Ida (prepared or others). From group comparisons, caregivers caring for someone with mild dementia were less likely to report disaster preparedness. Caregivers with higher levels of response efficacy, having an emergency plan in place, and with lower levels of avoidance toward death tended to report disaster preparedness. From logistic regressions, spousal caregivers were 4.46 times more likely to report disaster preparedness. Caregivers with lower levels of avoidance toward death were 1.52 times more likely to report disaster preparedness. Caregivers with higher levels of response efficacy were 2.36 times more likely to report disaster preparedness. This study offers theoretical and practical implications on how to engage and support Chinese American communities in disaster planning.

